# Guiding through: RALF signaling during the pollen tube growth in maize

**DOI:** 10.1093/plcell/koae024

**Published:** 2024-01-24

**Authors:** Nitin Uttam Kamble

**Affiliations:** Assistant Features Editor, The Plant Cell, American Society of Plant Biologists; Biochemistry and Metabolism Department, John Innes Centre, Norwich Research Park NR4 7UH, UK

Successful plant reproduction, crucial for yield and food production, is intricately regulated by pollen germination and pollen tube growth, processes highly sensitive to temperature. Angiosperms have evolved pollen tubes that carry their passive and immobile sperm cell cargo deep into the maternal reproductive tissues toward the embryo sac for double fertilization (Dresselhaus et al. 2016). Autocrine signaling pathways regulated by rapid alkalinization factors (RALFs) play a crucial role in hydration as well as cell wall integrity during pollen tube growth, a mechanism essential for double fertilization. As pollen tubes navigate from stigma to ovules, they secrete proteins, including RALFs, important for communication with maternal tissues. RALFs have been extensively studied in *Arabidopsis* (Zhong et al. 2022) but remain unexplored in cereal crops such as maize.

In this issue of *The Plant Cell*, **Liang-Zi Zhou, Lele Wang, Xia Chen, and colleagues** (Zhou et al. 2023) used gene expression data and phylogenetic analysis to identify candidate orthologs of RALFs in B73 maize inbred lines. Through genetic and biochemical studies, they demonstrated the involvement of RALF orthologs in pollen germination and tube growth. This study focused on two RALFs of clade IB and two RALFs of clade III of a total of 24 RALFs genes in maize. Using GFP-tagged ZmRALFs, the authors observed distinctive localization patterns in that only clade IB ZmRALF2/3 located to the cell wall, while clade III ZmRALF1/5 were found in the ER and cytoplasmic vesicles. Functional analysis using *ZmRALF* RNAi and CRISPR-Cas9 indicated that clade IB RALFs regulate pollen tube integrity, with their downregulation leading to tube burst at the sub-apical region, in contrast to corresponding *Arabidopsis* RALF mutants that burst directly after germination and at the very tip of the pollen tube. The authors suggested that this result may be due to the differential accumulation of esterified pectin in the sub-apical region of WT and *ZmRALF* RNAi-mutant pollen tubes and the difference in growth rate between the two species. The authors also observed significant differences in cell wall structure and thickness between WT and *ZmRALF* mutant pollen tubes. Subsequently, they found that clade IB ZmRALFs can partially complement *Arabidopsis ralf4/19* mutant, while Clade III ZmRALFs could not rescue the pollen germination burst phenotype in these lines.

Zhou et al. further teased out molecular mechanisms by identifying maize-specific receptor-like kinases (ZmFERLs) and maize pollen-specific LORELEI-like GPI-anchored proteins (ZmLLGs) as candidate co-receptors using gene expression data and phylogenetic analysis. Protein-protein interaction assays using pull-down and microscale thermophoresis (MST) assays with *E. coli* purified proteins, with correct folding confirmed using dynamic light scattering (DLS) and nano-differential scanning fluorimetry (nanoDSF), demonstrated that clade IB ZmRALFs interact with pollen-specific ZmFERLs (Fig.). The authors identified maize pollen-specific LORELEI-like GPI-anchored proteins ZmLLG1/2, demonstrating the auxiliary role of ZmLLG2 in interacting with clade IB ZmRALFs. Additionally, this research also indicated a role for pollen-specific ZmPEX cell wall proteins in competing with ZmFERLs for ZmRALF binding (Fig.). Previous structural studies of this complex in *Arabidopsis* showed that the N-terminal domain of clade IB RALF peptides folds into bioactive, disulfide bond-stabilized proteins that bind LRX proteins to form an LRX8–RALF4 complex (Moussu et al. 2020). In a more recent study, it was also revealed that RALF4's polycationic surface specifically interacts with demethylesterified pectins in the LRX8-RALF4 complex, regulating pollen tube wall integrity and expansion (Moussu et al. 2023).

**Figure.**
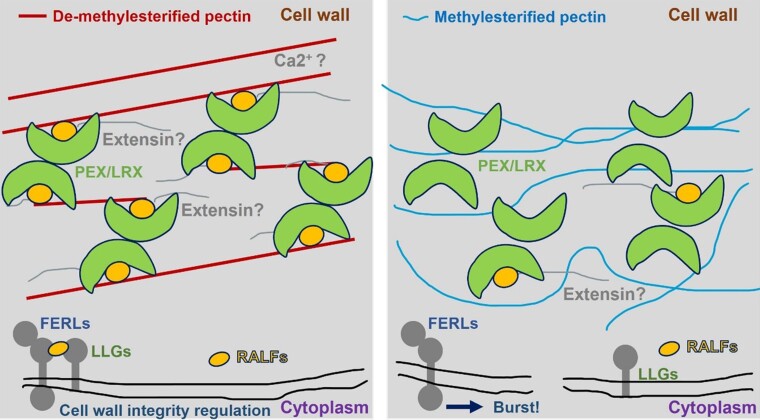
Maize clade IB ZmRALF signaling plays a crucial role in cell wall integrity. Zhou et al. (2023) found that mutation of RALFs results in the inactivation of ZmFERLs-ZmLLG1/2 signaling, with dilated cell walls due to fewer dimers. This affects interactions with methylesterified pectins, causing pollen tubes to burst at the sub-apical region. Figure credit: N. Kamble.

In summary, this research sheds light on the intricate molecular mechanisms underpinning autocrine RALF signaling in pollen tube growth and cell wall integrity and shows that the classical RALF signaling pathways also operate in cereal crops, in particular maize, to regulate pollen tube growth. Further work is needed to explore the roles of other RALF clades, their interactions with co-receptors (LLG) and cell wall proteins (LRX/PEX), and whether RALFs serve dual roles as sensors and cell wall components. Understanding these mechanisms has implications for improving fertility and thus food security worldwide.

## Data Availability

No new data were generated or analysed in support of this research.
